# Screening for endometriosis: A scoping review of screening measures that could support early diagnosis

**DOI:** 10.1186/s12905-025-03866-1

**Published:** 2025-07-16

**Authors:** Brittany N. Rosenbloom, Tania Di Renna, Adriano Nella, Mathew Leonardi, Maggie Tiong, Seungmin Lee, Rachael Bosma

**Affiliations:** 1 Women’s College Hospital, Toronto, ON Canada; 2 University of Toronto, Toronto, ON Canada; 3 McMaster University, Hamilton, ON Canada; 4 Institute for Work and Health, Toronto, ON Canada; 5 76 Grenville Street, Toronto, ON M5S 1B2 Canada

**Keywords:** Endometriosis, Scoping review, Screening measure, Validity, Patient Reported Outcome Measure (PROM)

## Abstract

**Background:**

Endometriosis is prevalent in approximately 6–10% of all women of reproductive age and is associated with pelvic pain, heavy menstrual bleeding, infertility, and pain during intercourse. Despite reporting symptoms, women wait around 11 years before receiving a diagnosis, further interfering with their mental and physical health. Patient reported screening measures can promote faster diagnosis, however their measurement quality remains unknown. Our objective was to identify and assess the measurement properties of endometriosis screening tools in a clinical setting.

**Methods:**

We searched Medline, Embase, and CINAHL from January 2010 until February 15th, 2024, as well as the reference list of all included studies. Two reviewers independently assessed eligibility at all stages of the review. Study quality was assessed with a modified COSMIN framework and stoplight system in which the measurement properties of each Patient Reported Outcome Measure (PROM) were ranked and scored as positive (green), negative (red), or unknown (yellow).

**Results:**

Of the 6082 studies that were collected, 24 were assessed for eligibility and eleven PROMs met our inclusion criteria and had their data extracted. A majority of the included studies assessed very few measurement properties (e.g., measurement error, structural validity, construct validity or responsiveness, etc…) of the PROM, leaving their quality unknown. The ENDOPAIN-4D received a positive rating in six out of ten measurement properties, ranking highest among the included studies. A Machine Learning Algorithm (MLA) developed by Bendifallah et al. (2022) also received good content and criterion validity, however required both patient report and clinical indicators.

**Conclusion:**

Of the included PROMs, the ENDOPAIN-4D was found the be the highest quality and could be adopted for a primary care setting. While the MLA could be used in a tertiary or specialist care setting reliance on more advanced data. However, like most studies included, the scope of its application is limited due to the potential homogeneity of ethnicity, gender, and socioeconomic status of the sample.

## Introduction

Endometriosis is a systemic, inflammatory condition characterized by the presence of endometrial-like tissue outside the uterine cavity [1]. It is prevalent with approximately 6–10% of all individuals assigned female at birth within reproductive age globally [2]. Common symptoms include painful menstruation (dysmenorrhea), heavy menstrual bleeding, severe non-menstrual pelvic pain, pain during intercourse (dyspareunia), painful defecation (dyschezia), and infertility [3]. Symptoms can present as early as an individual’s first menstrual cycle in adolescence and maintain until or even after menopause [3]. The presence of persistent pain, in combination with the lack of a curative treatment, can result in lifelong mental and physical health complications that severely reduce quality of life [3, 4].

Women can wait up to 11 years before receiving a diagnosis [5–7], and many report seeing a doctor at least five times before being referred to a specialist to receive a diagnosis [8]. It is estimated that 6 of 10 endometriosis cases remain undiagnosed, leaving many to struggle with cyclical or chronic pain and infertility without the proper treatments to aid in symptom management [7]. The non-specific symptoms of endometriosis overlap with other gastrointestinal or gynecological diseases, as well as the requirement for surgical confirmation make a definitive diagnosis challenging [3, 9]. Delays in diagnosis are costly, contributing to disease progression, higher likelihood of fertility problems, significant patient distress arising from diagnostic uncertainty, the need for more complex intervention, and increased healthcare utilization costs [4, 9–11].

A reliable screening tool for endometriosis could enable earlier detection, reducing diagnostic delays and minimizing complications such as chronic pain, infertility, and disease progression [12]. Recent advances in symptom-based screening measures centered on the description of symptoms according to patient-reported experience have been shown to be useful, non-invasive, inexpensive aids in the diagnosis of endometriosis [7, 13]. However, to date, the preoperative use of questionnaires to support the diagnosis of endometriosis remains scarce, partially due to the lack of consensus regarding the quality of the patient reported screening tools [14]. Thus, the purpose of this scoping literature review was to identify screening measures currently available for endometriosis, assess their measurement properties (reliability, validity), identify potential gaps, and provide an overview of their applicability to implementation in clinical practice settings.

## Methods

### Eligibility criteria

Study design followed the Preferred Reporting Items for Systematic Reviews and Meta-Analyses Extension for Scoping Reviews (PRISMA-ScR) [15]. The review protocol was registered on the Open Science Framework database (osf.io/54pm6) [16]. The eligibility criteria for included studies are outlined in Table 1.

**Table 1:** PICOS inclusion and exclusion criteria

Criteria	Included	Excluded
Population	Symptoms or a diagnosis consistent with endometriosis Women	Studies involving only surgical, imaging, or biomarker diagnosis of endometriosis
Intervention and comparators	No specific intervention Focus on screening for detection/diagnosis of endometriosis	Studies that examined interventional outcomes only
Outcome	Symptom-based questionnaires/ measures, screening tools	Predictive models
Study design	Any study design that developed/assessed screening measures of endometriosis	Commentaries, editorials, reviews

### Search strategy

The search strategy was developed with support from a research librarian and included Medline, Embase, and CINAHL database searches from 2010 until February 15th 2024. A combination of medical subject heading terms and free-text terms was used in the searches (Table 2). Considered studies included those: (1) published and peer reviewed (unpublished manuscripts, reviews, commentaries, guidelines, protocols are excluded), (2) written in English (non-English studies were excluded due to limited resources to translate other languages), (3) published in 2010 or later, and (4) conducted in humans aged 18 or older.

**Table 2:** Final medline search strategy, conducted February 15, 2024 (limits: humans; adults; english language)

1. Disease terms	Endometriosis.mp. or exp Endometriosis
	Pelvic Pain/ or exp Pelvic Girdle Pain/ or exp Pelvic Floor Disorders/ or pelvic.mp. or exp Pelvic Floor/ or exp Pelvic Inflammatory Disease
2. Screening instruments	Diagnosis/ or exp Diagnosis, Computer-Assisted/ or exp Missed Diagnosis/ or exp Delayed Diagnosis/ or exp Early Diagnosis/ or exp Diagnosis, Differential/ or diagnosis.mp.
	measurement.mp. or exp Pain Measurement/
	“surveys and questionnaires”/ or health care surveys/ or patient reported outcome measures/ or health surveys/ or patient health questionnaire/ or self report/

### Study selection

Study titles and abstracts, followed by full-text reviews for studies that met the screening criteria, were reviewed independently by two of the three members of the research team (RB, BR, AN). If a disagreement arose during any stage of the screening process, a consensus meeting was held to resolve the discrepancy. In addition to the database search, a secondary search of the reference list from all included studies was conducted to find eligible studies not initially found within the database search. The titles and abstracts of potentially eligible studies found through the reference list search were screened by the research team (RB and AN) and the full-text studies were reviewed for inclusion (BR and RB).

### Inter-rater reliability methods

To ensure consistency and reliability throughout the screening and data extraction phases, an inter-rater reliability assessment using Cohen’s kappa statistic was calculated. For the screening stage, the expected agreement was calculated based on the marginal probabilities. For the data extraction stage, an expected agreement value of 50% was used. Kappa values range from − 1 to 1, in which a score of 1 indicates perfect agreement, 0 indicates agreement no better than chance and less than 0 indicates agreement worse than chance. Prior publications suggest kappa values above 0.75 are considered excellent, while values below 0.4 are considered poor [17].

### Data extraction

Data extraction was conducted for all included studies by a member of the research team (AN) and verified by a separate member (SL). Discrepancies were recorded to conduct a concordance analysis (inter-rater reliability), and disagreements were resolved by consensus through consultation with a third member of the research team (BR). Information extracted from the eligible studies include: study title, lead author, publication year, country, study setting, funding, sample size, participant age, participant gender, participant characteristics (e.g., body mass index, smoking), pain characteristics, co-morbidities, socioeconomic status, ethnicity, missing data, methods, construct evaluated, patient partners, measurement properties, authors interpretation of the results, limitations, conclusions, and potentially eligible references.

### Study quality and assessment

A modified COnsensus-based Standards for the selection of health Measurement Instruments (COSMIN) framework was used to assess the measurement properties of each screening measure and score each study (Table 3) [18]. The COSMIN guidelines assess measurement sufficiency across 10 properties, including structural validity, internal consistency, reliability, measurement error, cross-cultural validity, content validity, construct validity, criterion validity, responsiveness, and interpretability. The quality of the measurement property is extracted and ranked. A positive rank is given if it the evidence meets or exceeds the minimum criteria for sufficiency, a negative rank is given if the screening measure fails to meet the sufficiency criteria, and an unknown rating is given if the authors fail to address the measurement property. We then developed a stoplight system to assign a color depending on the quality of the measurement property. Green represents studies that received a positive rating value. Red represents studies that failed to reach a positive rating and yellow represents studies that we could not determine due to missing information. Table 3 describes the adapted COSMIN framework scoring as well as the stoplight system.

**Table 3:**
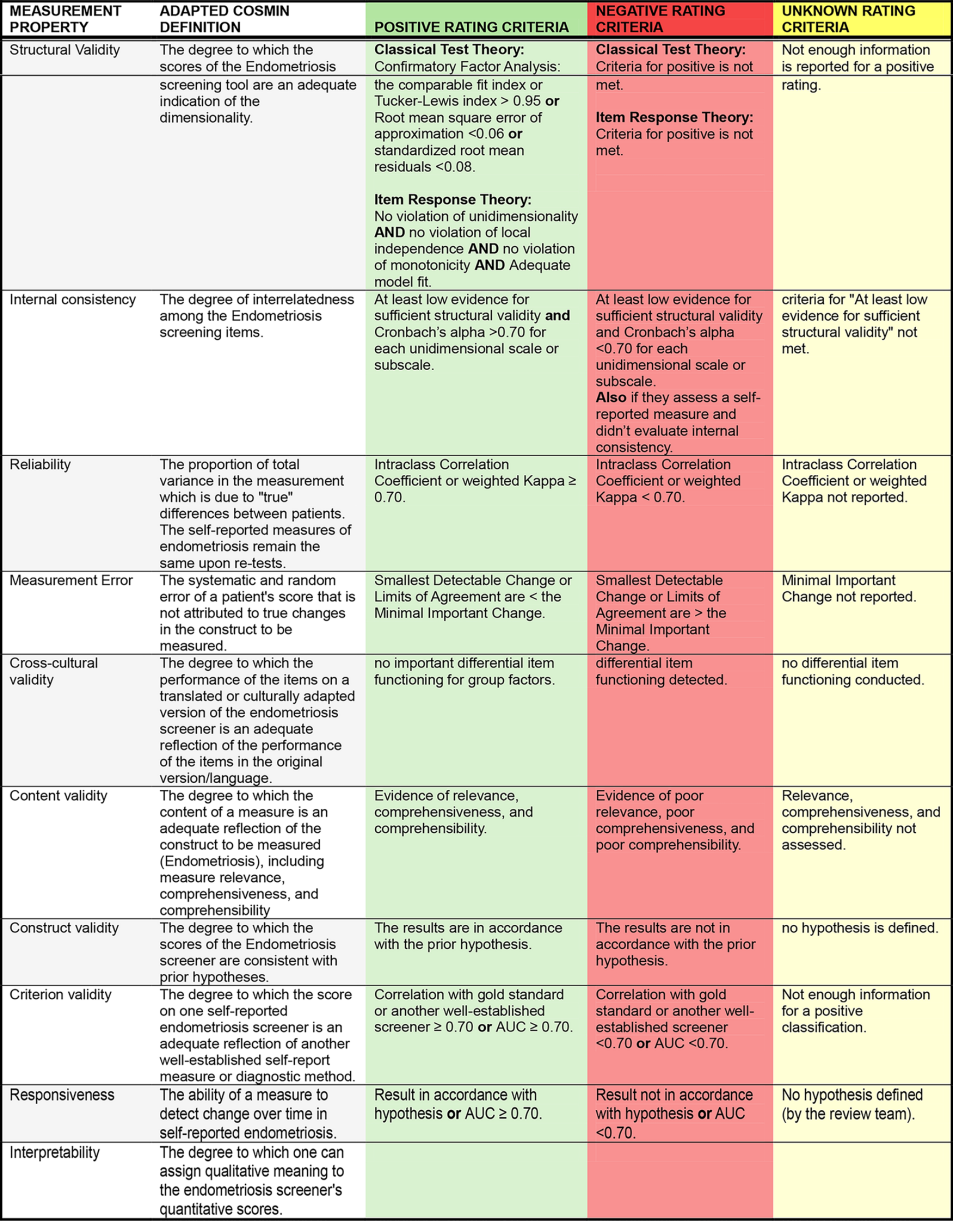
Stoplight system guidance based on COSMIN framework. AUC, area under curve

Upon the completion of the data extraction and COSMIN scoring, two experts in the field (TDR and ML) reviewed the results with the research team (BR and RB) to identify the most applicable screening measure. TDR is an anesthesiologist and chronic pelvic pain specialist with 14 years of experience. ML is a minimally invasive gynecologic surgeon and sonologist with 8 years of experience. Further, this expert review process delineated which screening measure would likely be the most applicable for a primary care setting, and which screening measure could be used within a specialized healthcare setting to assess endometriosis disease severity.

## Results

### Study selection

**Fig. 1:**
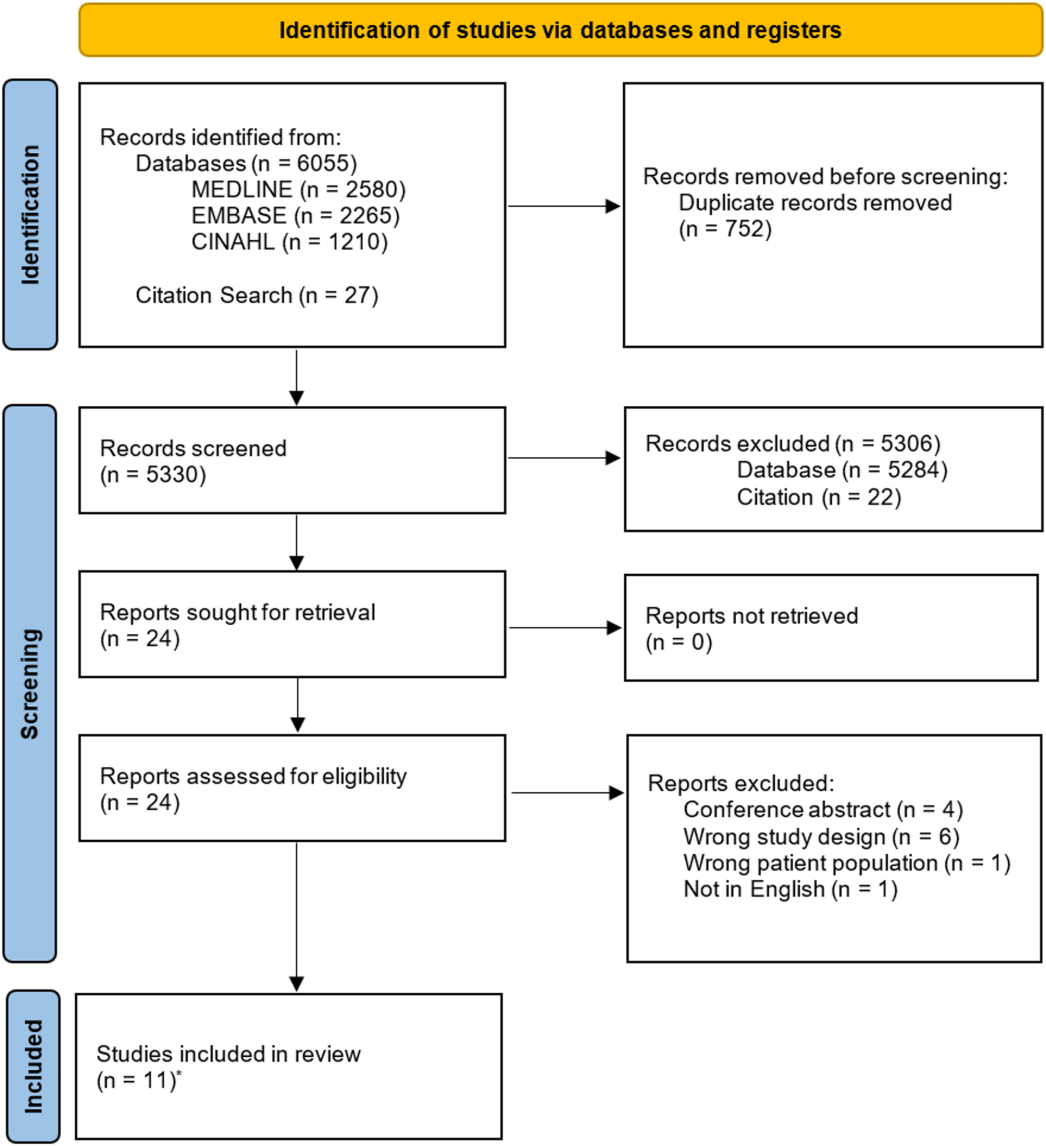
PRISMA flow diagram. *Two publications assessed the same outcome measure and were combined into one assessment for the extraction

A total of 6055 studies were identified via electronic database searches and 27 studies were identified via citation searching (Fig. 1). After removing 752 duplicate studies, a total of 5330 studies remained for title and abstract screening. A further 5306 studies were deemed ineligible, resulting in 24 full text articles being reviewed. Twelve articles met our inclusion criteria and were included for data extraction. Two of these articles featured the same endometriosis screening measure and were reviewed together for data extraction and summary, thus there were eleven unique screening measure studies extracted and reviewed.

### Concordance

The agreement was nearly perfect at the title and abstract range (~ 99%) and full text (89%). The concordance of the title and abstract screening phase ranged from 0.28 to 0.66, as indicated by the kappa coefficient. The full-text screening phase had a kappa value of 0.92.

### Study characteristics

Table 4 summarizes the details of the included studies. Of the eleven included studies, eight were conducted in Europe (France, Italy, Germany, and Norway), and three were conducted in the United States. Eight studies used self-reported patient questionnaires while the remaining three studies utilized machine learning methods to assign an endometriosis diagnosis. While all studies report the participant population being comprised of “women”, either during the recruitment methods or discussion of the results, no study included in this review analyzed the gender of the participants in further depth nor mentioned the inclusion of gender diverse populations. Explicit statements on how gender was assessed was not included in any study. Similarly, socioeconomic status and mental health co-morbidities present within the patient population were not reported in any of the studies. Ethnicity was reported in just three out of eleven studies [19–21]. Of those that did include, one contained a sample consisting of only Caucasian Italians [21], one contained a sample of predominantly Caucasian (90%) participants [19], and the other contained six Caucasian, eight African America, and two Hispanic participants [20].

**Table 4:** Characteristics of extracted measures

Title	Author and year	Study Setting and Country	Study Population	Construct Evaluated
Development of a Symptom-Based, Screening Tool for Early-Stage Endometriosis in Patients with Chronic Pelvic Pain [18]	Yeung et al. 2014	Hospital USA	• *N* = 90 • Endometriosis (*N* = 70) • Average age = 29.23 yrs • Socio-economic status, and co-morbidities: N/A. • Ethnicity: • White = 81 (90%) • Other = 9 (10%) • Sex/Gender: Women	Early-stage endometriosis model. Study participants completed a preoperative questionnaire in English with questions on physical and demographic characteristics, medical and family history, symptoms, and quality of life.
A new validated screening method for endometriosis diagnosis based on patient questionnaires [21]	Chapron et al. 2022	Hospital Frane	• Derivation sample (*N* = 1685) • Internal validation sample (*N* = 842) • External validation sample (*N* = 308) • Average age (only reported for derivation sample: • Endometriosis = 31.5 yrs • Socio-economic status, co-morbidities, and ethnicity: N/A • Sex/Gender: Women/NA	Self-developed measure featuring a Visual Analog Scale from 0–10 assessing primary or secondary dysmenorrhea, dyspareunia, pain of gastrointestinal (GI) origin, pain from urinary tract symptoms, and non-cyclical chronic pelvic pain. Data on the characteristics of the menstrual cycles, family history of endometriosis, and parity and gravidity.
Development of the painful periods screening tool for endometriosis [19]	DiBenedetti et al. 2018	Qualitative research facilities USA	• Endometriosis (*N* = 11) • Average age = 30.2 yrs • Socio-economic status, and co-morbidities: N/A, IBS • Ethnicity: (37.5%) white, 8 African American (50%), 2 Hispanic (12.5%) • Sex/Gender: Women/NA	Patient reported outcome for endometriosis Painful Periods Screening Tool (PPST)
Development of a prediction model to aid primary care physicians in early identification of women at high risk of developing endometriosis: cross-sectional study [22]	Verket et al. 2019	Community setting Norway	• Endometriosis (*N* = 157) • Average age = 35.2 yrs • Socio-economic status, co-morbidities, and ethnicity: N/A • Sex/Gender: Women/NA	Patient reported outcome for endometriosis newly developed questionnaire
The ENDOPAIN 4D Questionnaire: A New Validated Tool for Assessing Pain in Endometriosis [23]	Puchar et al. 2021	Hospital France	• Endometriosis (*N* = 132) • Average age: • Initial = 33.4 (6.8SD) yrs • follow up = 34.9 (7.2SD) yrs • Socio-economic status, co-morbidities, and ethnicity: N/A • Sex/Gender: Women/NA	Patient reported outcome for endometriosis ENDOPAIN-4D.
Early identification of women with endometriosis by means of a simple patient-completed questionnaire screening tool: a diagnostic study(13)	Fauconnier et al. 2021	Hospital France	• Endometriosis (*N* = 105) • Average age: 33 yrs • Socio-economic status, co-morbidities, and ethnicity: N/A • Sex/Gender: Women/NA	Patient reported outcome for endometriosis ENDOPAIN-4D short form.
Case-control study to develop and validate a questionnaire for the secondary prevention of endometriosis [20]	Ricci et al. 2020	Hospital Italy	• Endometriosis (*N* = 51) • Average Age = 36.9 yrs • Ethnicity: Caucasian Italian • Socio-economic status and co-morbidities: N/A • Sex/Gender: Women/NA	Patient reported outcome for endometriosis. The questionnaire is divided into 8 modules, with 47 questions in total. The first module focuses on the responder’s knowledge of the disease. The issues of the other 7 modules concern physiological case history, family history, remote medical history, phenotypic traits, gastrointestinal symptoms, urinary symptoms, and, lastly, gynecological history.
Use of the Free Endometriosis Risk Advisor App as a Non-Invasive Screening Test for Endometriosis in Patients with Chronic Pelvic Pain and/or Unexplained Infertility [[24]	Nezhat et al. 2023	Not reported. USA	• Endometriosis (*N* = 276) • Average age = 35.79 yrs • Socio-economic status, co-morbidities, and ethnicity: N/A • Sex/Gender: Women/NA	Endometriosis Risk Advisor mobile application (EndoRA). AI-based algorithm that categorizes patients based on their chief complaint: infertility or pain. A series of questions focusing on symptomatology, family history, psychiatric history, past medical history, fertility issues, and prior fertility testing. The AI-based algorithm calculates the risk assessment as low risk (< 50%), moderate risk (50–75%), or high risk (> 90%) of possibly having endometriosis
Predictive Model for the Non-Invasive Diagnosis of Endometriosis Based on Clinical Parameters [14]	Konrad et al. 2023	Hospital Germany	• Endometriosis (*N* = 177) • Average age: 34.3 yrs • Socio-economic status, co-morbidities, and ethnicity: N/A • Sex/Gender: Women/NA	Self -developed patient reported questionnaire.
clinical score can predict associated deep infiltrating endometriosis before surgery for an endometrioma [25]	Pillet et al. 2014	Hospital France	• Endometriosis (*N* = 326) • Average age: 31.5 yrs • Socio-economic status, co-morbidities, and ethnicity: N/A • Sex/Gender: Women/NA	Self-developed questionnaire for deep-infiltrating endometriosis The patient’s questionnaire was a preoperative questionnaire completed at the first preoperative consultation by the surgeon in charge of the patient. It contained 57 variables, including: General data, Gynecological data, History of symptoms and treatments during adolescence, The characteristics of all pains, both menstrual and non-menstrual.
Endometriosis Index A software-derived score to predict the presence and severity of the disease [26]	Fasciani et al. 2010	Hospital Italy	• Endometriosis (*N* = 95) • Average age: 36 yrs • Socio-economic status, co-morbidities, and ethnicity: N/A • Sex/Gender: Women/NA	Unified evaluation model (endometriosis Index).
Machine learning algorithms as new screening approach for patients with endometriosis(27)	Bendifallah et al. 2022	N/A France	• Endometriosis (*N* = 1126) • Average age: = 39 yrs • Socio-economic status, co-morbidities, and ethnicity: N/A • Sex/Gender: Women/NA	Machine Learning Model to predict endometriosis.

### Quality assessment

Table 5 summarizes the measurement properties of the PROMs based on the modified COSMIN framework. Criterion validity was the only measurement property to consistently (10/11 studies) achieve a green light by meeting or exceeding the minimum criteria for sufficiency. Content validity was described in 5/11 studies and all other parameters either failed to meet the sufficiency criteria or were unknown across most studies (> 7/11). Yeung et al., 2014, Chapron et al., 2022, and Pillet et al., 2014 only assessed the criterion validity of their self-reported endometriosis questionnaire, and all received a positive rated with and AUC ≥ 0.70 (reported AUC of 0.822, 0.73–0.81, and 0.84 respectively) [19, 22, 23]. Fasciani et al., 2010 also only assessed criterion validity, and reported a sensitivity of 72.4% and a specificity of 90.1%, also receiving a positive rating. Nezhat et al., 2023 also assessed criterion validity of the EndoRA with a sensitivity of 93.1% and specificity of be 5.9% [24, 25]. The positive predictive value (PPV) of the EndoRA score was calculated to be 94.1%. Konrad et al., 2023 found a PPV of 0.874 with a sensitivity of 90.0% and specificity of 75.0% [14]. DiBenedetti, et al., 2018 conducted their assessment with consultation from the Endometriosis Patients’ Advocacy Board and received a positive rating for content validity but failed to assess internal consistency of the Painful Periods Screening Tool (PPST) [20]. Verket et al., 2019 conducted their study with the Norwegian Endometriosis Association with content validity and an AUC ≥ 0.83 [26]. Ricci et al. 2020 modified their measure with feedback from 20 patients, conducted a literature review to select content for their measure and received an AUC of 0.91 [21]. Bendifallah et al., 2022 featured endometriosis experts to develop the clinical assessments and received an AUC of 0.50–0.93 [27].

**Table 5:**
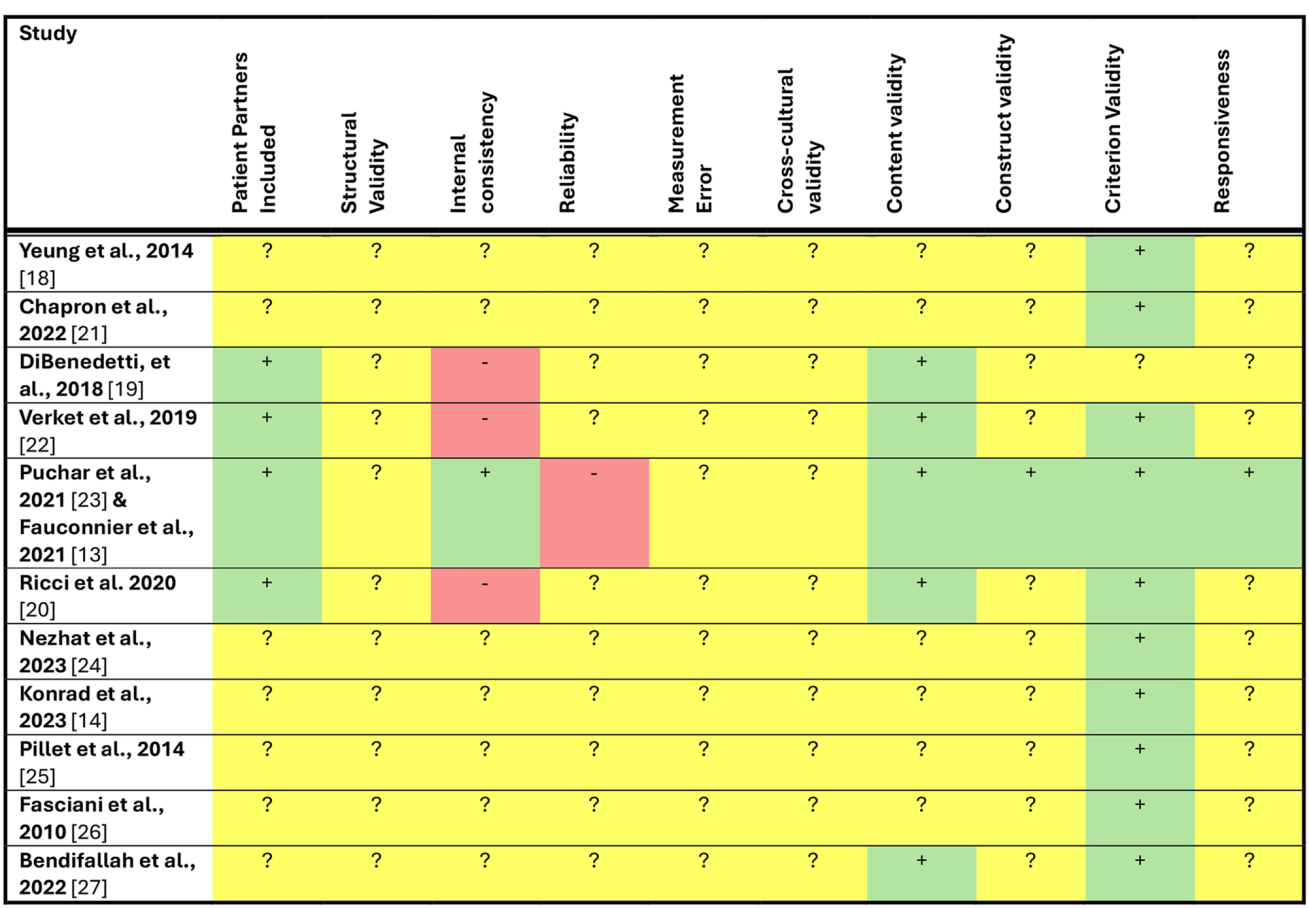
Stop-light rating system. Green represents studies that Met our rating criteria. Red represents studies that failed to reach a positive rating and yellow represents studies that we could not determine due to missing information

Puchar et al. 2021 and Fauconnier et al. 2021 assessed the validity of the ENDOPAIN-4D [13, 28]. The research was conducted in collaboration with Endo- France (patient partners), received a Cronbach’s Alpha of 0.79, 0.80, 0.83, and 0.77 (internal validity), was developed through a modified Delphi study (content validity), demonstrated high correlation with other patient-reported outcome instruments used for endometriosis patients (*p* < 0.05) (construct validity), received an AUC of 0.95 (criterion validity), and demonstrated statistically significant responsiveness for all sub scores (responsiveness). The ENDOPAIN-4D failed the assessment of reliability by scoring a weighted Kappa of 0.63.

### Synthesis

Consensus was reached between clinical experts (TDR and ML) on the first round of discussion yielding the same recommendations, which included: [1] ENDOPAIN-4D was the most robust screening measure of the identified screening measures and would be applicable for primary care; [2] a machine learning algorithm to diagnosis endometriosis by Bendifallah et al. 2022 provided the best measurement properties (content and criterion validity) and applicability for tertiary care.

## Discussion

This scoping review identified 11 screening measures described in 12 studies that pertained to endometriosis screening measures. Overall, based on the findings and after consultation with clinical experts, the ENDOPAIN-4D (screening measure) developed by Puchar et al. and the machine learning algorithm (MLA) developed by Bendifallah et al. (assessment of presence of endometriosis and disease severity) emerged as promising screening tools for further development and validation. The ENDOPAIN-4D’s validity indicates that it can be given to patients in primary care as a recommendation of further evaluation by a specialist (e.g., gynecologist). The MLA by Bendifallah et al. (2022) has good content and criterion validity and was designed for both assessing the presence of endometriosis as well as disease severity, which relies on both patient report and clinical indicators. Together the application of this MLA requires more advanced data gathering that is likely only available in tertiary care or with a specialist.

Development and implementation of standardized screening protocols in primary care remain underutilized but have the potential to enhance early detection, reduce the diagnostic delay, and ultimately improve patient outcomes by facilitating timely interventions and preventing the long-term complications associated with untreated endometriosis [3]. The screening tools identified in this review primarily used structured questionnaires to help healthcare providers systematically capture and evaluate symptoms such as chronic pelvic pain, dysmenorrhea and gastrointestinal disturbances. Several other screening tools also integrated advanced technologies, such as the MLA developed by Darai et al. and the AI-powered phone app (EndoRA) developed by Nezhat et al. [25, 27]. These tools are particularly noteworthy as they offer a free and accessible tool directly to patients, empowering them to actively engage in their healthcare. However, while advancements in algorithm-driven models and AI-powered tools are promising, these require computational resources that may limit their use in certain contexts. Additionally, developing screening measures must involve key users to ensure validity and relevance. All identified measures were developed in hospital settings, with only one including people with lived experience, and it is unclear if primary care clinicians were involved.

The development and validation of the identified endometriosis screeners raise several concerns. The findings reveal a predominant focus on criterion validity, assessed in 10/11 studies, and construct validity, assessed in 5/11 studies. Among the studies that assessed criterion validity, nine compared their screener to the gold standard of diagnosis — surgical histopathology. This emphasis on criterion validity is crucial for establishing diagnostic accuracy, while the focus on content validity ensures that the screeners comprehensively encompass all relevant aspects of endometriosis. However, there is a notable gap in the evaluation of other key psychometric properties. Other than the ENDOPAIN 4D questionnaire, which also assessed internal consistency, reliability and construct validity, all other screeners did not assess any other psychometric properties. In fact, three screeners did not report any evaluations for internal consistency despite it being a self-reported measure. The lack of cross-cultural validity assessments limits the global generalizability of these available screening measures. Measures identified in this review were created and validated primarily in Europe and cultural and linguistic differences can significantly influence symptom reporting and interpretation, underscoring the importance of validating screening tools in diverse populations [29].

The limited reporting of demographic characteristics in these studies exacerbates these concerns. Only 3/11 studies provided demographics data, with none reporting on patient socioeconomic status or the inclusion of gender-diverse individuals. This oversight is significant, as it risks perpetuating healthcare disparities. Historically, endometriosis has been perceived as a condition predominantly affecting affluent White women who postpone childbearing [30–32]. Although it is now recognized that endometriosis can affect individuals from all racial and ethnic groups, the prevalence is reported to be much lower among non-white women [33, 34]. The extent to which diagnostic biases and differences in clinical presentation contribute to this discrepancy remains unclear. Likewise, gender-diverse individuals were not included. Gender-diverse individuals may experience unique symptoms and barriers to diagnosis as they often face stigmatization which may deter them from seeking medical help, further complicating their healthcare outcomes [35]. Furthermore, the endometriosis screeners included were developed in older adults, typically aged 30–40, with more severe disease presentations. This neglects younger adult populations who may present with different symptom profiles and disease severities [36]. The lack of focus on younger populations in current research underscores a critical gap that must be addressed to ensure comprehensive and equitable healthcare.

### Limitations

While a comprehensive search strategy was developed with the aid of an experienced librarian, it remains possible that we did not capture all available and eligible research. Further factors that may have limited the scope of the search strategy is the lack of unpublished or grey literature as well as the English language restriction. The conclusions of our study may be limited by the scope of scoping reviews. Unlike a systematic review, no risk of bias assessment was conducted and thus some of the included studies may be of low quality. However, the modified COSMIN framework and stoplight system still provide extensive data on the validity of each measure to enable use within a healthcare setting. Furthermore, symptom onset for endometriosis may occur before adulthood, thus future work needs to be done to identify screeners developed for adolescents.

## Conclusions

In conclusion, twelve studies featured validated endometriosis screening tools. ENDOPAIN-4D [28], developed with patient input, and the MLA tool [27] were the highest rated for validity and effectiveness. Further research is needed to assess these tools in more diverse populations, including gender-diverse individuals, different ethnicities, socio-economic backgrounds, and younger groups.

## Data Availability

The dataset used and analyzed during the current study are available from the corresponding author on reasonable request.
